# Cerebral Hemodynamic Changes During Cardiac Surgery: A Feasibility MR Study in Piglets

**DOI:** 10.1002/nbm.70284

**Published:** 2026-04-05

**Authors:** Dominik T. Schulte, Ruth O'Gorman Tuura, Henning Richter, Michael Hofmann, Francesca del Chicca, Tobias Aigner, Christoph Loeschmann, Martina Lentini, Manuela Wieser, Frauke Seehusen, Rima Bektas, Melanie Zeilinger, Martin O. Schmiady, Marianne Schmid Daners

**Affiliations:** ^1^ Institute for Dynamic Systems and Control ETH Zurich Zurich Switzerland; ^2^ Centre for MR Research Children's Hospital Zurich Zurich Switzerland; ^3^ Diagnostic Imaging Research Unit, Vetsuisse Faculty University of Zurich Zurich Switzerland; ^4^ Centre for Cardiac Surgery, University Hospital Zurich University of Zurich Zurich Switzerland; ^5^ Anesthesiology Animal Hospital Zurich Zurich Switzerland; ^6^ Institute of Veterinary Pathology, Vetsuisse Faculty University of Zurich Zurich Switzerland

**Keywords:** animal model, cardiac surgery, cardiopulmonary bypass, cerebral perfusion, perisurgical MRI scans, white matter injury

## Abstract

Congenital heart disease often requires early surgical intervention. Cardiopulmonary bypass (CPB) is a standard procedure, but infants undergoing CPB show an increased risk of postoperative white matter injury. These injuries are associated with adverse neurodevelopmental outcomes, yet the underlying mechanisms remain poorly understood. MR imaging offers unique opportunities to study cerebral perfusion and metabolism in vivo but has not previously been feasible during CPB with a heart‐lung machine (HLM) in close proximity to the patient and the MR scanner, respectively, because conventional HLMs interfere with scanner operation. To address this limitation, we developed an MR‐conditional HLM and evaluated it in an in vivo piglet model, which closely mimics the neonatal brain and circulation. Unlike conventional systems, our MR‐conditional roller pump can be positioned directly inside the scanner bore without compromising imaging quality or pump performance. Four trials were conducted, in which piglets were cannulated and maintained on CPB inside the MR scanner. During CPB, cooling, rewarming, and unilateral carotid clamping were performed while repeatedly acquiring MR data on flow, diffusion, perfusion, and metabolism. Crucial brain structures—including the basal ganglia, hippocampus, and internal capsule—were consistently visualized without artifacts, and no disturbances occurred in pump operation or monitoring signals. The MR scans revealed flow‐related changes and a progressive increase in lactate, suggesting the onset of metabolic stress under hypoperfusion. These results demonstrate the technical feasibility of performing CPB within the MR scanner using an MR‐conditional HLM. Although not sufficient to identify specific mechanisms of brain injury, this platform provides a unique foundation for mechanistic studies in relevant large‐animal models. In the future, such an approach could help identify early markers of cerebral vulnerability, improve the safety of pediatric cardiac surgery, and inform the development of neuroprotective strategies for CPB and extracorporeal membrane oxygenation patients.

## Graphical abstract

Congenital heart disease surgery in infants requires cardiopulmonary bypass (CPB), which increases the risk of brain injury. Conventional heart‐lung machines (HLMs) cannot operate inside MRI scanners, limiting real‐time assessment of cerebral changes. We developed an MR‐conditional HLM and tested it in piglets during CPB. The system enabled continuous MR imaging of perfusion, diffusion, and metabolism, revealing hypoperfusion‐related changes and rising lactate. This establishes the feasibility of combining CPB with MRI for mechanistic brain studies.

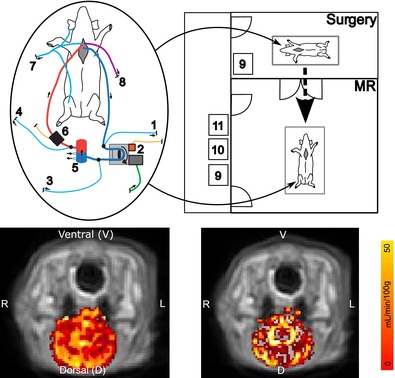

**Abbreviations**
CHD: congenital heart disease
CPB: cardiopulmonary bypass
HLM: heart‐lung machine
PCB: printed circuit board

## 1 Introduction

Cardiopulmonary bypass (CPB) is an essential component of cardiac surgery in neonates, particularly for the correction of congenital heart defects (CHD) such as single ventricle defects [1]. During such surgery, the CPB temporarily replaces the functions of the heart and lungs with an extracorporeal support [2]. The blood is drained from the body—most commonly via the superior or inferior vena cava—and returned to the aorta, though alternative cannulation sites may be utilized based on the patient's anatomy [3]. Although CPB is a well‐established technique in adults, with over 2000 procedures performed daily worldwide [4, 5], its neonatal application presents additional challenges: very small blood volume, immature organ systems, and elevated risk of neurologic injury. Neonates with congenital heart disease often show abnormal brain development even before surgery [6]. Recent reviews and guidelines on neonatal and infant CPB underscore further factors, such as the need to optimize oxygen delivery, manage hematocrit, minimize inflammatory responses, and use perfusion strategies tailored to immature physiology, that distinguish neonatal CPB from adult practice [7, 8, 9].

Neonates with congenital heart defects often present with white matter brain injuries even before surgery, with reported incidences increasing by 20%–50% following cardiac surgery with CPB [10, 11, 12]. This raises concerns about the effects of CPB on cerebral perfusion and oxygenation. However, research by Bertholdt et al. [13] showed a reduction in white matter brain injuries by a few days postsurgery compared to preoperative levels, suggesting complex interactions between surgery, CPB, and neonatal brain recovery. Accurate and comprehensive monitoring of cerebral dynamics during CPB is, therefore, crucial to understand and mitigate potential brain injuries.

MRI is a key diagnostic tool for detecting brain injuries and assessing structural, functional, and metabolic changes in the brain. However, the ferromagnetic components of conventional heart‐lung machines (HLMs) prevent their use near MRI systems. Because the HLM provides and controls circulation during CPB, its integration into the MR environment is essential to enable real‐time monitoring of cerebral changes directly during surgery. Without such integration, important insights into how CPB affects the brain, such as alterations in perfusion, metabolism, and white matter integrity, cannot be captured, creating a significant gap in understanding the mechanisms underlying perioperative brain injury. Studies by Spielman et al. [14] and Mutch et al. [15] show approaches of investigating the interaction of CPB and brain metabolism by placing the pump system outside of the MR room. However, neonates' limited blood volume, typically around 300 mL assuming 85 mL/kg [16, 17], further complicates the design of any study investigating MR‐conditional HLMs, as tubing length and extracorporeal blood volume must be minimized. Other groups placed the actuation parts outside of the MR room and transferred the rotational energy to the pump head using long poly‐carbonate drive shafts [18] or a flexible drive cable [19], both resulting in substantial spatial requirements. To address these challenges, a custom‐built MR‐conditional HLM has been developed previously, designed to eliminate ferromagnetic components and minimize electromagnetic interference with imaging. Careful material selection, shielding of the sensor systems, and spatial separation of electronic parts from the MR‐suite were required to ensure that the pump operated reliably without image distortion. This MR‐conditional roller pump can provide a continuous, nonpulsatile flow of up to 2798.20 ± 10.40 mL/min, while causing a reduction in SNR of −8.43% ± 7.96% [20]. The hemolytic behavior of the pump has been investigated in a nonphysiological pressure head of 10 mmHg, where the pump showed similar behavior to a LivaNova Sorin Stockert S5 (SOMA TECH INTL, Bloomfield CT, United States) [21].

The primary aim of this study was to evaluate the performance of a custom‐built MR‐conditional HLM during MRI. A piglet model is used due to the physiological similarities between piglets and human neonates, providing a robust preclinical model for studying hemodynamic changes and brain injury mechanisms [22, 23]. In this study, piglets larger than those representative of neonates or infants were used, primarily because smaller animals posed technical limitations such as insufficient vessel diameter for safe cannulation, difficulty in accommodating the MR‐compatible circuit, and challenges in maintaining adequate blood volume for CPB support. The custom bypass integrates sensors to measure pressure, flow, temperature, and pump speed, enabling real‐time data acquisition and visualization. A secondary aim is to investigate the effects of pump flow on cerebral hemodynamic and metabolism during CBP, assessed with a comprehensive multimodal MRI and MRS protocol.

## 2 Methods

### 2.1 General Experimental Setup

The in vivo study was conducted at the Vetsuisse Faculty, University of Zurich. All animal experiments were approved by local governmental authorities (License No. ZH161/2022) and conducted according to the Swiss Animal Welfare Act. Duroc piglets (*n* = 11), aged 6–8 weeks and weighing 13–15 kg, were selected for the experiments due to their comparable physiological characteristics to humans [24]. Different trial groups were defined based on standard procedures commonly used in cardiac surgery, namely, unilateral perfusion, full body perfusion, and deep hypothermic cardiac arrest [25, 26]. For the current study, a total of *n* = 4 animals is presented, of which two belong to the unilateral perfusion group and the other two to the full‐body perfusion group. The deep hypothermic cardiac arrest group was excluded due to deviations from the intended experimental conditions. In one trial, excessive bleeding during the trial caused the animal to suffer from hemorrhagic shock, in the other trial dysfunction of electrical equipment limited our data recording capabilities. Both groups investigated targeted a body temperature of 28°C; however, in the full‐body perfusion group, blood circulation remained unchanged, whereas in the unilateral perfusion group, the right carotid artery was clamped to modify blood flow distribution.

### 2.2 Anesthesia

General anesthesia was induced using a tight‐fitting face mask and a prefilled circle system (7% Sevoflurane in 100% oxygen) with an advanced breathing system at a flow rate of 4 L/min. Adequate spontaneous breathing was monitored using capnography and end‐tidal CO_2_ (ETCO_2_). End‐tidal Sevoflurane concentrations during mask induction were 5.00% ± 0.50%. Intravenous access for medication was then established through the caudal auricular veins, and an arterial line was placed in the femoral artery for intraarterial blood pressure monitoring. The piglet's trachea was intubated, and general anesthesia was maintained using Propofol (6 mg/kg/h), Midazolam (1 mg/kg/h), Ketamine (0.50 mg/kg/h), and Remifentanil (0.10 μg/kg/min) to maintain adequate plane of anesthesia and analgesia. The given rates were adjusted according to the depth of anesthesia (DoA). DoA indicators included motor response to a stimulus (nasal septum pinch, tail clamp, claw clamp, and reaction to painful/surgical stimulus), muscular tone, anal tone, corneal reflex, heart rate, and blood pressure. Pancuronium was administered as a bolus infusion at the start of the experiment (0.05 mg/kg) and then as a steady infusion (0.25 mg/kg/h) to provide muscle relaxation. During instrumentation, the piglets were first ventilated in synchronized intermittent mandatory ventilation pressure controlled before neuromuscular relaxation. Thereafter, pressure‐controlled ventilation was used until the surgical intervention made volume‐controlled ventilation necessary. Respiratory rate, peak pressure, and tidal volume were adjusted to maintain adequate ETCO_2_ but within normal physiological limits. The piglets were artificially ventilated to maintain arterial partial pressure of CO_2_ between 35 and 45 mmHg and hemoglobin saturation with oxygen of 98%–100%. The piglets received a 1% glucose containing ringers lactate infusion (10 mL/kg/h). Oxygenation during CPB was regulated while maintaining pH according to the alpha‐stat strategy, ensuring blood gasses were managed relative to the patient's temperature [27].

### 2.3 Surgical Procedure

A median sternotomy was performed to enable cannulation of the superior vena cava and central aorta [24]. In addition, the right carotid artery was exposed, and the vessel was slung to allow clamping without removing the animal from the MR scanner. A modified left ventricular vent was used in place of a conventional venous cannula, as MR‐compatible venous cannulas with the necessary wall reinforcement are not available. This cannula featured an open tip and side perforations and was therefore ideally suited for placement in the superior vena cava with the tip positioned close to the right atrium. Placement in the atrium itself was avoided due to the fragility of atrial tissue, where manipulation can result in tearing or trigger cardiac arrhythmias. The superior vena cava, in contrast, is structurally more robust and less prone to such complications, making it the preferred access site. However, it should be noted that if the vessel diameter is too small, the cannula can become obstructive, and the suitability of the vena cava must therefore be evaluated before use.

### 2.4 Experimental Process

**FIGURE 1:**
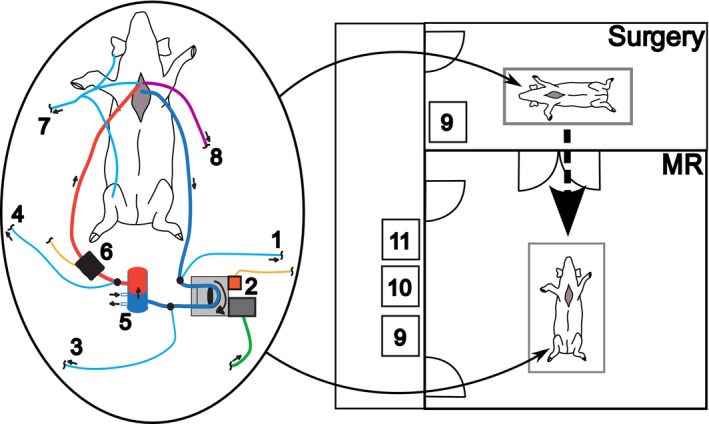
Schematic overview of the experimental setup. The two main locations indicate where anesthesia and cannulation were performed (“surgery”) and where the cardiac surgery is emulated (“MR”). Blood is drawn from the body via the vena cava, with a pressure sensor (1) detecting negative pressure to prevent suction before the MR‐compatible pump (2). Additional pressure sensors (3, 4) monitor pressure before and after the oxygenator (5), whereas a flow sensor (6) measures blood flow directed toward the animal. Another pressure sensor (7) monitors arterial blood pressure. The thoracic placement of the temperature probe (8) is marked in purple. As the animal is transported to the MR room, anesthesia pumps (9) are relocated outside. The hypothermia device (10) and the main electronics of the measurement system (11) are positioned near the waveguides outside the MR room.

The experimental process was divided into two main phases: preparation outside of the MR room and testing within. The preparation phase included anesthesia administration and cannulation. These steps were performed outside the MR room, as most surgical tools are not MR safe. Once the cannulation was completed, the tubing was first connected to a Sorin Stockert S5 (LivaNova, London, United Kingdom) roller pump, which was mounted on a Sorin Stockert SCP (LivaNova, London, United Kingdom) frame for reduced spatial requirements. The target pump flow is calculated according to the body weight of the animal [28]. Once the animal was stable, the tubing was switched over to the MR‐conditional roller pump. Following preparation, the animal was transported to the MR room and put into the scanner. During the transfer, most sensors and medication lines were temporarily disconnected, whereas their cables and tubes were threaded through the waveguides into the scanner room. An overview of the infrastructure and setup is provided in Figure 1.

Once the animal was positioned inside the scanner, a survey MR scan was performed to correctly plan the measurement planes for subsequent scans. As many structural and functional baseline scans as feasible were then acquired before initiating the cooling process, to minimize the time between the start of measurements and the onset of cooling. Cooling was then initiated, and additional MR scans were acquired, with a focus on spectroscopy to monitor changes in brain temperature throughout the cooling process. Upon reaching the target body temperature of 28°C, additional experimental modifications, such as clamping, were implemented. The animal was maintained at the target hypothermic state for approximately 30 min, mimicking the duration of CHD aortic arch repair surgery. Due to spatial constraints within the MR scanner and the lack of suitable MR‐conditional surgical instruments, no surgery was performed beyond the cannulation procedure. After 30 min, the body temperature was gradually increased to normothermic conditions (approximately 38°C). The experiment concluded with the euthanasia of the animal. After euthanasia, the brain was removed and fixed in 10% buffered formalin at an approximate ratio of 1:10 (specimen: formalin) for 48 h. Following fixation, various brain regions—including different areas of the cerebrum, cerebellum, and brainstem, as well as multiple aspects of the ventricular system—were carefully trimmed in coronary sections. The tissue samples were then embedded in paraffin, and hematoxylin–eosin–stained sections were prepared according to standard laboratory protocols.

### 2.5 Technical Setup, Electronics, and Software Interface

To enable measurements during the experiments, sensors were attached to the animal and the CPB system (Figure 1). The encoder (ME22‐LD, PWB encoders GmbH, Eisenach, DE) was shielded in copper to reduce scanner‐induced RF interference because it is mounted to the MR‐conditional roller pump. An oxygenator (Paragon Adult Oxygenators, Chalice Medical Limited, Worksop, United Kingdom), mounted downstream of the pump with a custom fixture, was connected to an oxygen supply and two hypothermia device water tubes. The circuit included four pressure sensors (Meritrans DTX Plus, Meritmedical, South Jordan UT, United States) connected via three‐way connectors. During surgery, sensors were placed near the animal to minimize signal damping, but inside the MR scanner, they remained outside the Faraday cage due to ineffective shielding solutions. One sensor monitored pressure inside the venous cannulation line before the pump to detect suction events, whereas sensors before and after the oxygenator monitored the pressure gradient to detect possible coagulation and blockage. The fourth sensor was used to monitor arterial pressure of the animal. Arterial pressure was measured at different sites depending on the animal, with the cannulation location selected based on individual anatomical accessibility.

A flow sensor (Sonoflow CO.55, Sonotec, Halle, DE), encased in custom copper shielding, measured pump‐generated flow immediately downstream of the oxygenator. A temperature sensor (Luxtron 812 Fiber Optic Thermometer, Advanced Energy, Denver CO, United States) placed outside the Faraday cage utilized a glass fiber probe inserted into the rectum or thorax to monitor core body temperature.

The electronics are integrated into the system via a custom‐designed printed circuit board (PCB) that interfaces with a Microlabbox (DS1202, dSpace, Paderborn, DE). The PCB houses amplifier circuits and hardware filters designed to amplify the weak sensor signals to measurable levels, while also filtering out noise caused by the MR scanner's radiofrequency signal. Signals from inside the Faraday cage are routed through a waveguide, where a custom hardware filter (high‐density DSub Filter, RF Immunity, Yavne, Israel) absorbs radiofrequency interference, ensuring only the filtered signal reaches the PCB. A 24‐V power supply unit powers the PCB.

A computer running dSpace ControlDesk (Version 7.0 Release 2019‐A, dSpace, Paderborn, DE) with custom MATLAB Simulink (Version R2018b, Mathworks, Natick MA, United States) serves as the operational hub, visualizing, monitoring, and recording sensor data with a sampling frequency of 200 Hz. Two monitors facilitate operations: one near the surgical site and another one by the hypothermia device. Pump operation is controlled through a dedicated controller box, consisting of a potentiometer to adjust speed and a switch for immediate on/off control. The controller box is connected to the Microlabbox via a long cable, allowing the pump to be controlled directly from the surgical site during cannulation as well as from the hypothermia device.

The software focuses on real‐time visualization and monitoring. Each sensor's data are displayed in individual plots, with the corresponding moving average (calculated over a 10 s window) displayed alongside the plots. Pressure sensors are assigned configurable standard ranges, with colored LEDs indicating whether values fall within these customizable limits (e.g., 40–70 mmHg). Optional audio alarms can alert deviations from these thresholds.

Rotational pump speed is managed via a hand‐tuned digital proportional‐integral‐derivative feedback controller also running at 200 Hz. Any deviations of the pump speed to the setpoint are highlighted by an LED and an optional audio alarm. A critical safety function prevents tissue damage by automatically reducing pump speed if upstream pressure drops below a customizable threshold, indicating potential vena cava tissue suction. An audio alarm is triggered, and the pump speed reduces to 25% of the current setpoint. Once the pressure rises above another customizable threshold, the suction event is deemed to be resolved, and the speed is restored to 100% of the setpoint. This safety mechanism, along with both adjustable thresholds, can be controlled via the user interface.

### 2.6 MR Image Acquisition

MR morphological and functional images were acquired. All scans were performed on a Philips Ingenia Elition X (Philips, Amsterdam, NL) 3 Tesla scanner equipped with a dedicated 32‐channel coil head coil (dStream HeadNeck, 32ch, Philips AG Healthcare, Horgen, CH). Specific parameters are listed in Table 1.

**TABLE 1:** Parameters of the different MR sequences, including repetition time (TR), echo time (TE), field of view (FoV), acquisition matrix (AM), reconstruction matrix (RM), and slice thickness (ST). Some parameters were adapted to the animal (a.t.a).

Name	TR/TE (ms)	FoV (mm^2^)	AM	RM	ST (mm)	Additional parameter
Turbo spin echo T2‐weighted	2000–7993/80–100	a.t.a	168–236 × 104–155	288 × 288 to 528 × 528	1.5	—
3D turbo field echo T1‐weighted	13/5.9	a.t.a.	168 × 167	240 × 240	0.6	—
CINE gated phase‐contrast	12/7.4	150 × 150	256 × 179	256 × 256	4	Encoded velocity 120 mL/min
Diffusion tensor	3822/91	a.t.a.	104 × 102	128 × 128	2	*b* = 800 s/mm^2^ 32 encoding directions
pCASL	4171/13	170 × 170	96 × 96	64 × 60	4	—
PRESS	2000/288	—	—	—	—	Voxel size 10 × 20 × 27.54 mm, 160 averages, readout duration 512 ms

Morphological brain images were acquired with Turbo Spin Echo T2‐weighted imaging in the transverse, sagittal, and dorsal planes. The 3D Turbo Field Echo T1–weighted sequence was acquired on the sagittal plane.

Blood flow dynamics in major vessels were assessed using PC imaging. The sequence was acquired with the imaging plane perpendicular to the carotid arteries and jugular veins at the level of the occipital‐atlanto joint, and flow through these vessels was measured. The gating probe of the MR scanner was placed on the tubing of the CPB after the pump and triggered according to the pulses induced by the roller pump instead of the pulsation induced by the heartbeat.

DTI was employed to assess the diffusion of water molecules in the brain tissue. Temperature dependence of diffusion is evaluated by fitting a linear function to the data points for cooling and heating. Perfusion measurements are obtained using pCASL. Metabolic data were acquired using single voxel PRESS.

### 2.7 Data Processing and Evaluation

The digital signals, sampled at 200 Hz, from the pressure, flow, and temperature sensors, as well as the encoder, are processed for analysis. Signals from the pressure and temperature sensors are filtered using a moving median filter with a window size of 100 s. The encoder signal is converted into rotational speed via the dSpace Software's Encoder module and subsequently filtered using a moving median filter spanning 20 pump rotations. To prevent the visualization of inactive measurements after system shutdown, the recordings are terminated at pump shutdown, even if additional MR sequences are still being run.

The MR morphological images and histopathology were analyzed by experienced veterinary specialists in diagnostic imaging and pathology (Diplomate ECVDI and EVDP, respectively) to identify any visible lesions or tissue damage in the brain.

The PC images were processed using the MEDIS QFlow software (Version 8.1, Medis Medical Imaging Systems, Leiden, NL), employing the built‐in *QFlow* module. Regions of interest (ROIs) included the carotid arteries, jugular veins, and collateral veins. The ROIs were initially defined using the software's automatic center‐click function, which detected the vessel boundaries. These boundaries were manually adjusted frame by frame if the automatic segmentation missed segments of the visually assessed vessel. The software calculated flow per frame and provided the average flow for each ROI. The total average flow through both carotid arteries was summed to assess the amount of blood flow diverted toward the brain. Similarly, the total average flow through the jugular and collateral veins was summed to assess the venous outflow from the brain.

The DTI scans underwent preprocessing with eddy current correction to correct for distortions caused by motion or eddy currents. Diffusion tensors were subsequently fit using the *dtifit* function of the FMRIB Software Library (FSL) (Version 6.0.2). To analyze diffusion in specific brain regions, the brain atlas for 12‐week‐old piglets developed by Fil et al. [29] was registered to the data. This registration process was performed using the *ANTSregistration* function of Advanced Normalization Tools (ANTs) (Version 2.1.0‐gGIT‐N). The registered atlas was then applied as a mask to extract the average diffusion metrics, such as fractional anisotropy and mean diffusivity, from specific brain regions (basal ganglia, hippocampus, cortex, and thalamus) within the DTI images. The measurements are then visualized in relation to the corresponding thresholds for each region.

The pCASL scans were analyzed using a ROI‐based approach with the same piglet brain atlas. Due to the low resolution and SNR of the pCASL images, direct registration of the atlas using the *ANTSregistration* function was unsuccessful. Instead, the atlas fitted to the DTI scans was resampled and aligned with the pCASL images. This approach ensured consistency in ROI extraction across modalities, allowing the same brain regions to be evaluated for perfusion and diffusion changes. Cerebral blood flow was quantified in milliliters per 100 g of brain tissue per minute (mL/100 g/min).

The data acquired from the PRESS scans were processed using the linear combination of model spectra (LCModel, Version 6.3‐1R) method to quantify the levels of key metabolites, including choline, NAA, and lactate, as ratios to creatine within the voxel [30]. Due to the temperature dependence of the water peak, the LCModel peak referencing was performed using the main metabolite peaks (NAA, Cr, and Cho). The ratios between lactate/creatine and NAA/creatine were calculated and reported for each measurement. Linear regression was used to calculate the slope per hour of the NAA/creatine ratio. Additionally, the resonance frequencies of water, choline, creatine, and NAA were utilized to calculate the brain temperature [31, 32]. The temperature was determined as an average of the three formulas introduced by Zhu et al. [33], which calculate temperature based on the differences in peak frequency between water and choline, water and creatine, and water and NAA, ensuring robust and reliable estimation of cerebral temperature dynamics during the experiments. The frequency shifts between the water and metabolite peaks were measured with jMRUI version 5.2 [34].

## 3 Results

A total of four trials were conducted, referenced in the following as Animals A–D. The timestep labeled as “0 h” corresponds to the start of the first MR scan, marking the point at which all measurements are initiated and can be compared. Surgery started 1 h 35 min before the first MR scan for Animal A, 2 h 16 min for Animal B, 1 h 54 min for Animal C, and 2 h 9 min for Animal D. The first two animals (A and B) were part of the full body perfusion group and the second two (C and D) of the unilateral perfusion group. In the experiment with Animal A, the MR scanner's gating device failed to detect flow pulsations, preventing carotid arterial flow measurements. For Animal C, severe blood loss during and after surgery caused the superior vena cava to collapse, resulting in continuous suction and visible air embolism in the tubing, ultimately leading to the early termination of the experiment.

**FIGURE 2:**
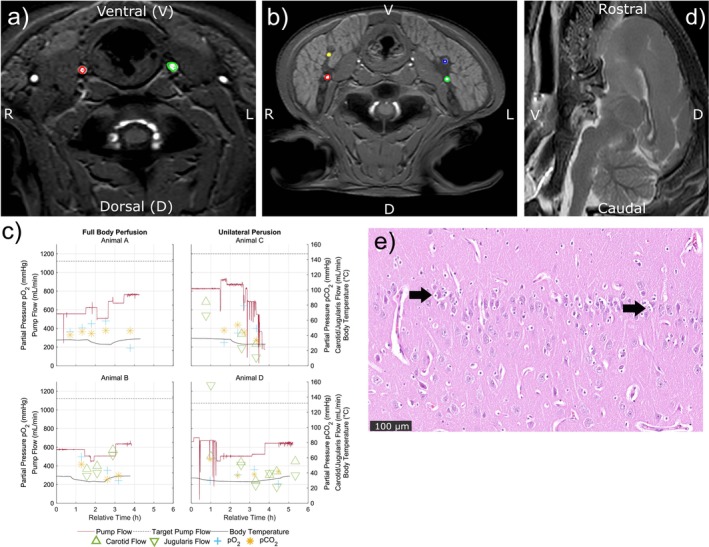
Example placements of regions of interest for phase‐contrast flow measurements in the carotid arteries (a) and the jugular (red and green) and collateral veins (blue and yellow) (b). (c) Measured flows from these vessels alongside the generated and target pump flows, body temperature, and partial pressures of O_2_ and CO_2_ over time. An example sagittal T2‐weighted MR image of the brain is shown in (d), whereas (e) depicts neurons of the cerebral cortex with minimal increase in perineuronal glial cells (satellitosis), visualized with hematoxylin and eosin staining.

Figure 2 illustrates the placement of regions of interest (ROIs) for phase‐contrast imaging in the carotid arteries (Figure 2a) and in the jugular and collateral veins (Figure 2b). Figure 2c presents the blood flow generated by the pump alongside the measured flows in these vessels across all animals. Sudden drops in pump flow, represented by vertical lines, indicate automatic system responses to suction events. The data confirm that the MR scanner's radiofrequency signals did not interfere with sensor performance, ensuring reliable measurements. Morphological MR images (Figure 2d) appeared normal with no visible lesions or artifacts. Postmortem histopathological analysis (Figure 2e) showed no signs of hemorrhage or lesions; however, a minimal multifocal activation of glial cells was noted in the form of minimal neuronal satellitosis in the cerebral cortex.

In Animal A, pump flow was gradually increased throughout the experiment. However, due to the lack of cardiac gating, the carotid and jugular flow could not be assessed. Although pCO_2_ levels remained stable, pO_2_ levels dropped from 479.40 to 189.80 mmHg. The maximum pump flow was 772.92 mL/min (55.21 mL/min/kg). Animal B exhibited a continuous decline in pO_2_ levels. During the cooling phase, pump flow was reduced, with carotid and jugular flow closely mirroring these changes. The maximum pump flow was 669.00 mL/min (47.79 mL/min/kg). The trial with Animal C faced significant suction issues, resulting in stronger‐than‐usual flow disturbances and frequent safety interjections. Despite stepwise reductions in flow to compensate for blood loss, stability was not achieved. Carotid and jugular flow steadily declined, whereas pO_2_ levels increased overall, and pCO_2_ levels gradually decreased. The maximum pump flow was 870.97 mL/min (58.06 mL/min/kg). Animal D also experienced initial suction issues (steep drops before and after 1 h of testing), but reducing the flow stabilized the cardiovascular system. This allowed for a gradual flow increase later in the experiment, only encountering flow fluctuations again toward the end of the trial. The maximum pump flow was 802.46 mL/min (59.89 mL/min/kg). Carotid flow, which initially declined slowly, dropped suddenly to 29.75 mL/min at 3 h 20 min after the start of the MR scans before recovering to 55.27 mL/min at 5 h. Jugular flow showed an exceedingly high flow of 155.68 mL/min in the first measurement before dropping to 50.57 mL/min.

**FIGURE 3:**
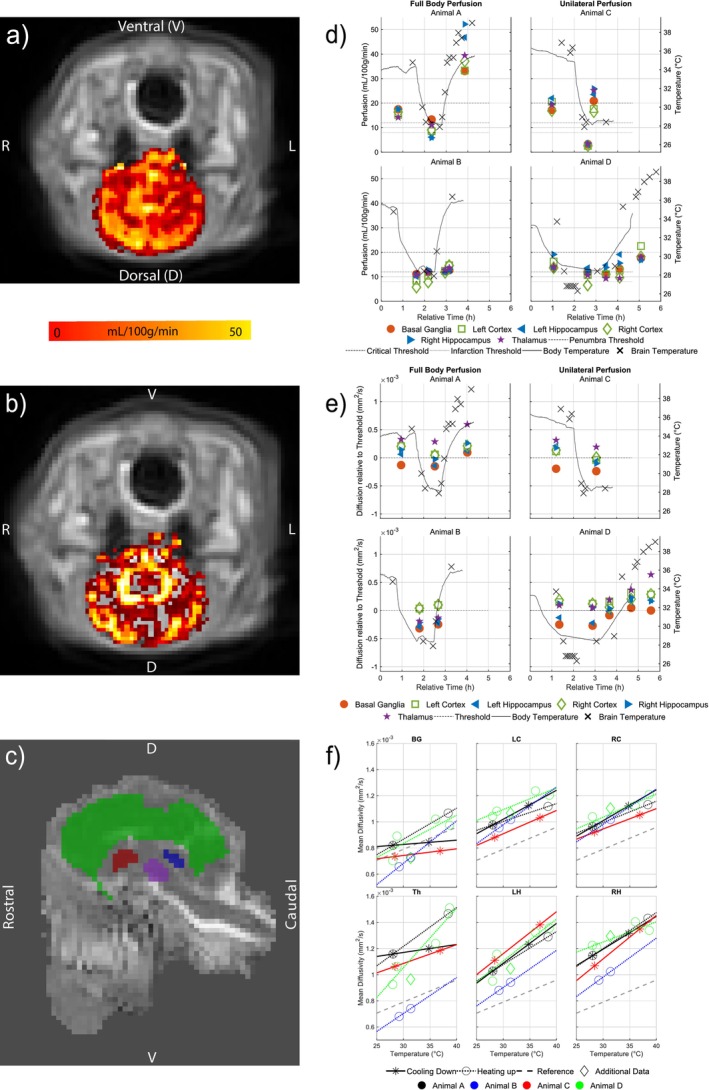
Example measurements from pCASL scans acquired early in the experiment (a) and after body temperature reduction (b). An example diffusion tensor imaging scan (c) illustrates the placement of regions of interest: cortex (green), hippocampus (blue), thalamus (purple), and basal ganglia (red). Measured perfusion values (d) are shown in absolute values of mL/min/100 g together with literature‐reported thresholds, whereas diffusion measurements (e) are displayed as values relative to their respective thresholds. Corresponding body and brain temperatures are also plotted. Panel (f) shows the relationship between brain temperature and diffusion for each region: basal ganglia (BG), left/right cortex (LC/RC), left/right hippocampus (LH/RH), and thalamus. Linear fits were applied to data collected during cooling (solid lines, stars) and heating (dotted lines, circles). A diamond symbol indicates a data point excluded from both fits. The dashed gray line represents the 2%/°C slope reported by Priest et al. [35] for comparison.

Figure 3 summarizes the results of perfusion and diffusion measurements. In Animal C, a reduction of blood flow in some regions, such as the cortex and luxury perfusion in others (particularly cortical and basal ganglia regions), was observed when comparing the first (Figure 3a) and last (Figure 3b) perfusion maps pre and post cooling. Figure 3c also shows the defined ROIs: cortex (green), hippocampus (blue), basal ganglia (red), and thalamus (purple) on the DTI map.

Figure 3d presents perfusion values in these ROIs, alongside ischemic thresholds reported in the literature [36]. A marked drop in perfusion follows cooling from ~35°C to 28°C, with partial recovery to above the measured baseline perfusion after rewarming. Brain temperature mostly tracked body temperature within few degrees, except during rewarming, where Animals A and D exhibited a more pronounced temperature overshoot. In contrast, Animal B showed a consistent offset before cooling, which may be attributed to the use of a thoracic rather than rectal temperature probe, resulting in closely matched brain and body temperatures throughout.

Figure 3e displays diffusion values in the ROIs, normalized to literature‐based thresholds rather than given as absolute values [37, 38, 39]. Similar to the perfusion measurements, diffusivity changes correlate with body temperature fluctuations, decreasing during cooling and increasing with rewarming. The differences in diffusion between left and right cortex and hippocampus, respectively, are small and differences between regions remain more or less constant over time.

Figure 3f illustrates the relationship between brain temperature and diffusion measurements, with linear fits applied to data from the cooling and heating phases. The linear relationship (2%/°C) between ADC and body temperature found by Priest et al. [35] is also included for comparison. For Animal C, data could only be fitted over the cooling phase, Animals B and D only over the heating phase. For Animal A, both fits are available. The slopes in the cortex displayed the least variability between animals and heating or cooling phases, ranging from slopes between 1.30%/°C and 2.93%/°C. Overall the slopes ranged between 0.39%/°C and 4.70%/°C.

**FIGURE 4:**
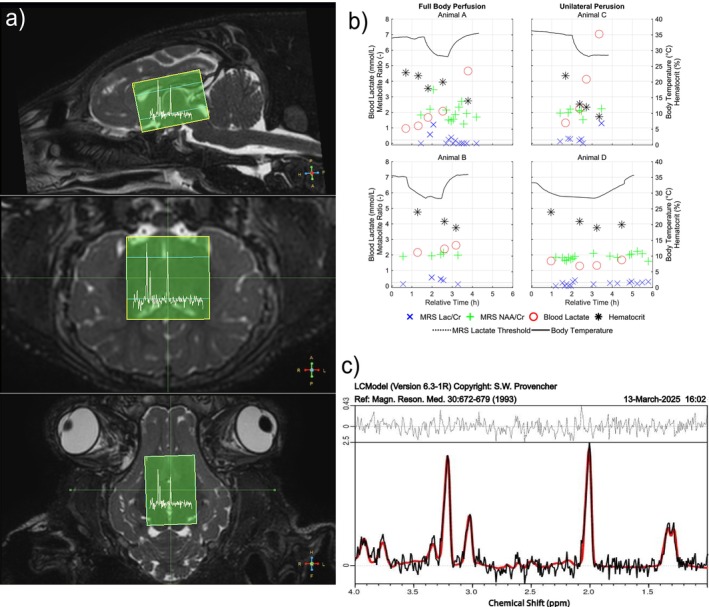
Placement of the measurement voxel for the single voxel point‐resolved spectroscopy scan in the sagittal, transverse, and coronal views (a). (b) Blood lactate levels and the ratios of spectroscopy‐derived Lac and NAA to Cr on the left *y* axis, alongside body temperature and hematocrit on the right *y* axis. A dotted line indicates the threshold for elevated lactate ratios. An example MR spectrum from the measurement is shown in (c).

Figure 4a illustrates the placement of the MR spectroscopy voxel centered on the thalamus, shown in both the sagittal, transverse, and coronal views. Figure 4b presents the time courses of lactate and NAA levels, shown as ratios to Cr, alongside blood lactate concentrations, hematocrit values, and body temperature. A dotted line indicates the threshold for elevated lactate ratios. Although blood lactate levels remained below 10 mmol/L across all animals, increases in both blood and MRS lactate were observed. Notably, Animal C showed a sharp lactate spike, peaking at 7.04 mmol/L. A gradual decline in NAA was observed in Animal A, where the slope of the linear regression over all measurements revealed a reduction in ratio of −0.2353 per hour. The slopes for the regression over the NAA/Cr ratios were 0.0491 (Animal B), 0.0569 (Animal C), and 0.0517 (Animal D) per hour. An example MR spectrum is shown in Figure 4c.

## 4 Discussion

Congenital heart diseases often require immediate surgical intervention shortly after birth, necessitating the use of CPB to temporarily replace heart and lung function. Although CPB is well established in adults, studies suggest it may contribute to white matter injuries in infants [10, 11, 12]. A key limitation of these studies is that MR imaging to assess brain injury could only be performed before and after surgery. In our previous publication, we introduced an MR‐conditional HLM [40]. This study presents the technical setup for its first in vivo application and preliminary findings from the experiments to gather insights into the dynamics inside the brain during CPB.

### 4.1 Feasibility and Function of the MR‐Conditional HLM

Before addressing physiological outcomes, we first confirm that the custom‐built HLM performed as intended within the MR environment. The technical setup successfully maintained heart and lung function for an extended period without interfering with MR scans or experiencing disturbances from the strong magnetic field, time‐varying gradient fields, or radiofrequency interference, even when positioned close to the scanner. The main differences to other published studies are that our complete pump system could be placed near the MR scanner, eliminating the need for long tubing [14, 15]. Because the pump is actuated with a pneumatic motor, no long drive shafts [18] or flexible drive cables [19] had to be used. The device reliably recorded measurements, and the pump effectively detected suction events, many of which were resolved automatically as the instantaneous reduction in pump speed released the tissue from the cannula.

### 4.2 Surgical and Cannulation Considerations

Variability in surgical start times, which spanned up to 40 min between animals, likely contributed to differences in physiological baselines at the onset of scanning. Such variability is representative of the natural differences observed between patients in clinical practice, including baseline perfusion, blood gasses, and hemodynamics. Although absolute baseline values may differ, all subsequent MR measurements were interpreted relative to each individual's baseline, ensuring that the trends observed in perfusion, diffusion, and metabolic changes remain informative for assessing the feasibility of the MR‐conditional HLM.

Venous drainage via the superior vena cava using a modified left ventricular vent further proved feasible and provided stable venous return throughout the experiments. This approach offered a practical solution in the absence of MR‐compatible venous cannulas, which typically require metallic reinforcement to prevent collapse under suction. Nonetheless, the method carries certain limitations, as the cannula may act obstructively in smaller animals with proportionally narrower vessels, underscoring the need for the development of dedicated MR‐compatible venous cannulas for future clinical translation.

### 4.3 Group Allocation and Experimental Design

Animals were assigned to either full‐body or unilateral perfusion groups to explore potential differences in cerebral response; circulatory arrest experiments were not included due to deviations from the intended experimental conditions, such as excessive bleeding during surgery or electrical equipment dysfunction, that limited data reliability. However, with only two animals per group, this study was not powered to detect statistically meaningful group‐level effects, and the results primarily reflect feasibility under the different experimental conditions.

Although our study utilized piglets aged 40–60 days (6–9 weeks), which are larger than human neonates, their brains remain in a phase of rapid growth and maturation. This developmental stage has been shown to approximate early human infancy rather than adulthood, making them a relevant preclinical model despite exceeding the strict neonatal period [41]. Longitudinal MRI studies demonstrate that piglets undergo accelerated postnatal brain growth between 2 and 24 weeks, closely paralleling human neurodevelopmental trajectories [42]. Thus, although our animals do not correspond exactly to term neonates, their developmental profile remains within a biologically relevant window for translation to early infancy in humans.

The overall study design resembles the study design presented by Spielman et al. [14] and Mutch et al. [15]. In our study, only the most essential procedures are done outside the MR scanner, in this case the cannulation, so that as much as possible can be observed through the MR measurements. Whereas Spielman et al. [14] focused mainly on recording metabolic changes with 1H MRS and Mutch et al. [15] focused mainly on T2‐weighted imaging, our study covers structural, flow, perfusion, diffusion, and spectroscopic measurements, with the downside of gaining less data per measurement mode, because only one scan can be conducted at a time.

Blood loss represented a major challenge across the experiments, as management inside the MR scanner was considerably more difficult than in a conventional surgical setting. In routine surgery, lost blood can be collected and recirculated using suction devices; however, such equipment could not be employed in the MR environment due to spatial and material constraints. Consequently, bleeding episodes required alternative management strategies and often led to hemodynamic instabilities of the piglets. This limitation was especially pronounced in Animal C, where excessive blood loss resulted in severe hemodilution and ultimately forced early termination of the trial. The findings from Animal C are treated as a case‐specific observation.

### 4.4 Morphological Imaging and Pathohistology

The primary method used by Beca et al. [10], Galli et al. [11], and Berthold et al. [13] was the assessment of structural T1‐ and T2‐weighted images. Morphological MR imaging failed to detect abnormalities, and only slight activation of glial cells in histopathology was present, which may indicate neuroinflammation. This aligns with the results of Coskun et al. [43] and Boichot et al. [44], who found that T2‐weighted imaging lacked prognostic value in the first 6–10 days after injury. A temperature‐dependent increase in T2 signal intensity during hypothermia as reported by Mutch et al. [15] could not be verified due to the low number of T2 scans. Accurate qualitative and quantitative evaluation of glial activation cannot be reliably achieved on the basis of hematoxylin and eosin overview staining alone. Comparative analyses of treated animals and age‐matched controls at the immunohistochemical level, using a panel of glial markers, require systematic morphometric assessments supported by an adequately powered cohort. However, the present study demonstrates that physiological MRI methods such as those measuring flow and perfusion, in parallel with spectroscopic measurements of lactate and NAA, can provide valuable information about brain physiology during CPB surgery.

### 4.5 Pump Flow and Hemodynamic Stability

The pump flow recordings indicate that the target flow rate based on body weight could not be maintained throughout the experiments. According to ELSO guidelines [28], the pump flow should be set to 80 mL/kg/min, translating to approximately 1150 mL/min depending on the animal's weight. However, achieving this flow was not possible due to frequent suction events, where the vena cava tissue adhered to the cannula. The maximum achieved flows ranged from 47.79 to 59.89 mL/kg/min (Animal A: 55.21 mL/kg/min; B: 47.79 mL/kg/min; C: 58.06 mL/kg/min; and D: 59.89 mL/kg/min). However, all were below the recommended guideline threshold of 80 mL/kg/min. To mitigate this, the flow setpoint was progressively lowered in an attempt to stabilize the animal. Whereas this approach was unsuccessful in Animal C, leading to early euthanasia, it effectively stabilized Animal D, allowing the experiment to be completed. Venous cannula obstruction due to vena cava collapse is a well‐documented issue and could potentially be mitigated in future studies by selecting an alternative cannula design [45].

### 4.6 Blood Loss and Hematocrit Changes

The primary cause of the vena cava collapse is presumed to be substantial blood loss during the trials. To counteract this, careful bolus infusions of Ringer's solution were administered, which consequently led to a reduction in hematocrit. This dilution‐induced anemia can result in symptoms such as tissue hypoxia and, in severe cases, hemorrhagic shock [46]. A corresponding drop in the partial pressure of oxygen is expected, as fewer red blood cells are available for oxygen transport.

### 4.7 Oxygenation Dynamics and Carbon Dioxide Regulation

Not all animals exhibited a noticeable drop in pO_2_. Arterial hyperoxia was maintained at the start of the intervention, following standard clinical practice to prevent hypoxic injury and minimize the risk of gaseous embolization [47]. This strategy also ensures sufficient oxygen availability when pCO_2_ rises, as elevated CO_2_ levels reduce hemoglobin's oxygen‐binding affinity [48]. The measured pCO_2_ levels remained near the target of 40 mmHg, as intended by anesthesia applied during the procedure. A temperature‐dependent decrease in pCO_2_ was particularly evident in Animals B and D, consistent with findings in the literature [49]. In contrast, Animal C exhibited a marked decline in pCO_2_ later in the trial, likely due to its exceptionally low hematocrit level of less than 10% [50].

pCO_2_ has been shown to influence cerebral perfusion, suggesting that carotid flow should correlate well with pCO_2_ levels [51]. However, our measurements did not reveal a clear relationship between pump‐generated flow, pCO_2_ levels, and carotid arterial flow. In two animals, jugular venous flow exhibited a consistent offset relative to carotid flow, as expected because the flow in the vertebral arteries was not considered. In Animal D, however, the relationship between jugular and carotid flow was highly inconsistent, likely due to improper placement of the measurement plane for the jugular veins.

### 4.8 Temperature Effects

Although rectal temperature is thought to reflect body and brain temperature, during the experiment, it became evident that fecal matter can confound the measurement of temperature with a rectal probe. Delays in cooling and heating led to observable lags between rectal and brain temperatures, particularly during the heating phase. In Animal B, where the temperature probe was placed inside the thorax, the brain temperature closely matched body temperature throughout the entire trial. These findings align with the consensus that brain temperature cannot be reliably predicted from body temperature alone [52, 53].

### 4.9 Perfusion Changes in the Brain

Quantitative perfusion maps provide insights into regional blood flow, with atlas regions enabling the assessment of average perfusion within specific regions of interest [54]. Because cerebral perfusion is influenced by both carotid blood flow and oxygen availability, reductions in either parameter are expected to impact brain tissue perfusion. Additionally, brain temperature regulation plays a critical role, as lower temperatures can reduce metabolic demand and alter blood flow dynamics. To further assess the structural and functional effects of hypoperfusion and ischemia in our study, perfusion parameters were analyzed in key brain regions, including the basal ganglia, thalamus, hippocampus, and cortex. Given the close relationship between perfusion and diffusion, these regions were also evaluated for changes in diffusion to identify potential indicators of cellular injury. Studies by Alderliesten et al. [37, 55] have emphasized the critical importance of assessing diffusion in the basal ganglia and thalamus, particularly in neonates experiencing perinatal asphyxia. Additionally, studies conducted shortly after birth demonstrate that ADC reductions are also evident in the hippocampus [39], a region critical for memory and learning. And lastly, the cortex, which plays a pivotal role in higher order functions, is also highly vulnerable to ischemic damage caused by altered perfusion [38, 56].

In our study, perfusion thresholds [36] were applied uniformly across all brain regions rather than being region specific. It is important to note that these thresholds are conservative, as they are based on normothermic conditions and identified several hours poststroke. Despite these limitations, they provide a useful reference for analyzing the data collected. The penumbra threshold of 20 mL/100 g/min indicates tissue at risk but potentially salvageable with timely intervention. The critical threshold of 12 mL/100 g/min signifies severely impaired perfusion, whereas the infarction threshold of 8 mL/100 g/min marks the point of likely irreversible damage. These values are well established in stroke and ischemia research in humans and serve as key benchmarks for assessing malperfusion and predicting neurological outcomes. Additional insights into cerebral oxygenation and hemodynamics could be gained in future studies by incorporating complementary monitoring modalities such as near‐infrared spectroscopy.

As expected, perfusion exhibited a general dependency on body temperature. Within each measurement, perfusion values remained relatively consistent across different brain regions, and no substantial differences were observed between full‐body and unilateral perfusion. In every trial, perfusion dropped below the infarction threshold in at least some regions; however, the perfusion consistently recovered with rising temperature or increased pump flow. In Animal C, a sharp increase in perfusion following a substantial drop below the infarction threshold is observed. It is reasonable to suspect an autoregulatory response, as the body attempted to preserve brain function in reaction to severely reduced flow and pO_2_ levels.

### 4.10 Diffusion Alterations

When perfusion is insufficient, the cellular energy metabolism is disturbed. This ultimately leads to an increase of Sodium ions in the intracellular space. Osmotic pressure then causes water to shift inside of the intracellular space. The swelling of cells (cytotoxic edema) and resulting decrease of extracellular volume can then by observed as a decrease in mean diffusivity (restricted diffusion) of water within the affected areas. The severity of this damage can be assessed through measurements of the ADC, as studies have shown ADC to be a reliable indicator of cellular integrity. Critical ADC thresholds have been identified in humans, below which cellular damage is likely: 973 × 10^−6^ mm^2^/s for the basal ganglia [55], 871 × 10^−6^ mm^2^/s for the thalamus [55], 1171 × 10^−6^ mm^2^/s for the hippocampus (associated with poor outcomes) [39], and 918 × 10^−6^ mm^2^/s for the cortex [38, 56]. These thresholds, established under normothermic conditions, like the perfusion thresholds, serve as conservative reference points for comparison in our study.

Because the ADC thresholds vary by region, the results are presented as values relative to their respective thresholds. As expected, diffusivity showed a clear correlation with body temperature. Consistent with the perfusion findings, no substantial differences between the left and right hemispheres of the hippocampus and cortex were observed, and diffusivity remained comparable in these regions. Unlike perfusion, diffusivity values did not consistently drop below the critical threshold in all regions, with the cortex largely remaining above threshold levels. The basal ganglia showed the most pronounced deviations, reaching a minimum of 657 × 10^−6^ mm^2^/s in Animal B. According to Alderliesten et al. [55], reduced ADC values indicate severe brain injury. However, due to the unaccounted influence of body temperature and other factors in our study, definitive conclusions regarding injury cannot be made.

### 4.11 Interaction of Diffusion With Brain Temperature

Another aspect that warrants further analysis is the rate of change in ADC in response to brain temperature fluctuations [57]. In our study, we specifically examined the ADC slopes during cooling and heating separately. Priest et al. [35] reported a 2.0%/°C decrease in ADC during cooling, which aligns closely with our observed values. Whereas the heating and cooling slopes in the cortex and hippocampus were comparable, significant deviations were noted in the basal ganglia and thalamus. As Alderliesten et al. [55] highlighted, these regions were particularly vulnerable to injury in perinatal asphyxia, suggesting that the altered temperature dependence of ADC may be linked to altered perfusion due to the pump flow not achieving the intended target. However, the reported decrease in ADC during cooling depends on many factors, technical related (e.g., length and way of cooling), and related to the subject (e.g., age and species).

### 4.12 Spectroscopy Findings

Another method for assessing brain injury is by evaluating lactate levels. Blood lactate has been identified as a reliable predictor of adverse outcomes in infants with hypoxic–ischemic encephalopathy due to perinatal asphyxia, with a reported cut‐off value of 14 mmol/L at 6 h postbirth [58]. In our study, the highest measured blood lactate level was approximately 7 mmol/L, suggesting a favorable outcome with no long‐term damage. However, the threshold for elevated lactate to creatine ratio is 0.22 [59], whereas ratios up to 1.29 were observed in Animal C. This discrepancy may arise because spectroscopy measures lactate not in blood but in tissue (cerebral gray and white matter) and cerebrospinal fluid. This aligns with findings by Shibao et al. [60], who reported no correlation between blood lactate and spectroscopy‐derived lactate levels in the cerebrospinal fluid.

Elevated lactate in spectroscopy measurements could indicate a metabolic shift to anaerobic glycolysis due to oxygen deprivation [61] or the use of lactate to aid with signal transduction during immune and inflammatory response [62]. Although the figures suggest that only minutes have passed since the start of surgery, they actually begin at the time of the first MR scan, so there was a substantial time (between 1 h 35 min and 2 h 16 min) before the first MR scan for insufficient perfusion to trigger anaerobic metabolism. A subsequent decline in spectroscopic lactate may indicate that the reduced body temperature lowers the metabolic demand, allowing a return to aerobic glycolysis and decreasing intracellular lactate levels. Spielman et al. [14] observed a similar behavior, where in their study during antegrade cerebral perfusion no buildup of lactate was observed as opposed to their observed increase during deep hypothermic circulatory arrest.

From the spectroscopy data, the levels of NAA can also be analyzed since a decrease in NAA is a well‐established marker of diffuse axonal injury or loss [63]. In our study, three out of four trials showed an overall reduction in NAA/Cr ratio during the experiment. A progressive decline in NAA is typically associated with ongoing injury or neuronal loss over an extended period [63] although reversible changes have also been observed in following transient middle cerebra artery occlusion [64]. Given the relatively short duration of our study, it remains unclear whether the observed reduction reflects irreversible damage or a transient metabolic response to the experimental conditions. Further investigation with longer observation periods would be necessary to determine the long‐term implications of these findings.

### 4.13 Study Limitations

The primary limitation of this study is the small sample size, with only four animals. This dataset allows for a qualitative assessment of the data, though further trials would be needed for comprehensive statistical analysis. Another major limitation was the considerable blood loss during cannulation. In all trials, blood loss necessitated bolus injections of Ringer's solution to compensate for the reduced blood volume, which otherwise led to vena cava collapse, suction events at the venous cannula, and a reduction in hematocrit. During the in vivo experiments, bleeding became a challenge inside the MR scanner, as the constrained space did not allow for recirculation of shed blood back into the circuit. Own‐blood donation could have helped maintain a more physiological hematocrit with potentially reduced impact of hemodilution and improved physical condition of the piglets. For the MRS measurements, no correction for T2 effects was performed [32], so the changes in NAA/Cr or Lac/Cr could be confounded by T2 changes to one or more of these metabolites. Lastly, the perfusion and diffusion thresholds are extrapolated from human medicine and therefore may not be directly applicable to the piglet brain, especially because tissue maturation of the pig brain differs substantially to the tissue maturation of an infant with similar body weight and age.

## 5 Conclusion

This study primarily demonstrates the feasibility of using an MR‐conditional HLM to investigate cerebral perfusion and metabolic changes during CPB. The MR measurements successfully captured reductions in perfusion, diffusion, and NAA, as well as an increase in lactate, which were associated with reduced pump flow. Dependencies between diffusion, perfusion, and both body and brain temperature were also identified. Although these findings confirm that the setup enables meaningful in vivo measurements during CPB, no clinically relevant conclusions can yet be drawn; instead, the study establishes feasibility and provides the groundwork for future investigations under more representative physiological and clinical conditions.

## Author Contributions

**Dominik T. Schulte:** hardware design, software design, conducting experiment, data analysis, writing manuscript. **Ruth O'Gorman Tuura:** study design, conducting experiment, data analysis, reviewing manuscript. **Henning Richter:** study design, conducting experiment, data analysis, reviewing manuscript. **Michael Hofmann:** study design, conducting experiment, securing funding, reviewing manuscript. **Francesca del Chicca:** conducting experiment, data analysis, reviewing manuscript. **Tobias Aigner:** conducting experiment, reviewing manuscript. **Christoph Loeschmann:** conducting experiment, reviewing manuscript. **Martina Lentini:** conducting experiment, reviewing manuscript. **Manuela Wieser:** conducting experiment, reviewing manuscript. **Frauke Seehusen:** data analysis, reviewing manuscript. **Rima Bektas:** conducting experiments, data analysis, reviewing manuscript. **Melanie Zeilinger:** data analysis, securing funding, reviewing manuscript. **Martin O. Schmiady:** study design, conducting experiment, securing funding, reviewing manuscript. **Marianne Schmid Daners:** study design, conducting experiment, data analysis, securing funding, reviewing manuscript.

## Funding

This study was supported by Innosuisse – Schweizerische Agentur für Innovationsförderung (56642.1 IP‐LS).

## Conflicts of Interest

The authors declare no conflicts of interest.

## Data Availability

The data that support the findings of this study are available from the corresponding author upon reasonable request.
